# Proposed Economic Threshold for *Diceraeus melacanthus* (Dallas) in Maize Under an Early Intercrop Cultivation System

**DOI:** 10.1007/s13744-026-01439-x

**Published:** 2026-09-16

**Authors:** Weidson Plauter Sutil, Letícia Carolina Chiampi Munhoz, Adeney de Freitas Bueno

**Affiliations:** 1 Dept of Biology, Federal University of Paraná, Curitiba, Paraná Brazil; 2 Postgraduate Program in Agronomy, State University of Londrina, Londrina, Paraná Brazil; 3 Embrapa Soja, Caixa Postal 4006, Londrina, Paraná 86001-970 Brazil

**Keywords:** Green-bellied stink bug, Antecipe®, feeding preference, sequential cropping

## Abstract

The Antecipe® system, in which maize is sown between soybean rows while soybean plants are still in the reproductive stage, reduces the production cycle of the soybean-maize crop system and, consequently, mitigate climatic risk for second-crop maize. However, the coexistence of both crops can intensify the ‘green bridge,’ favoring outbreaks of *Diceraeus melacanthus*. Thus, this study evaluated the host preference of *D. melacanthus* and the damage caused by different population densities to support management strategies in this system. The preference test was conducted in the greenhouse using cages (1.5 × 0.5 m) containing senescing soybean and maize at the VE stage, infested with ten adult *D. melacanthus*. Evaluations were carried out over three consecutive days at three times per day. The field experiment followed a completely randomized design with four stink bug densities (0, 1, 2, and 4 insects/m) in 6 × 4 m cages. When maize reached the VE stage, insects were introduced, and damage was assessed. Preference tests indicated a predominance of stink bugs on soybean, especially during the warmest periods of the day, whereas their activity on maize was higher in the early morning. In the field experiment, maize plants exposed to 2 and 4 stink bugs/m showed the highest damage levels, and yield reduction was observed at the density of 4 stink bugs/m; however, with 2 stink bugs/m, yield was statistically equal to both 1 and 4 stink bugs/m (intermediate yield), which could be a turning point from plant tolerance to plant compensation in the damage curve. Therefore, we propose that 2 stink bugs/m should be adopted as a more conservative (yet preliminary) economic threshold (ET) for maize cultivated in this system. Such ET must now be validated in farm conditions, under different climatic conditions (different regions), with different maize varieties.

**Supplementary Information:**

The online version contains supplementary material available at https://doi.org/10.1007/s13744-026-01439-x.

## Introduction

The soybean–maize succession is an important cropping system in the Neotropics and accounts for a substantial portion of grain production (Garcia et al. 2018). Brazil is the largest soybean producer worldwide, with ca. 47 million hectares cultivated during the 2024/2025, and within this production system, ca. 17 million hectares were allocated to second-season maize (*safrinha*) (Conab 2025). Despite its economic importance, maize cultivated in the second season is highly exposed to climatic risks (especially drought and frost), which are considered one of the main factors responsible for yield losses (Guimarães 2024).

In several Brazilian regions, the interval between soybean harvest (first season) and the establishment of maize (second season) has progressively shortened. This reduction is mainly associated with climatic variability, which delays either the soybean harvest or the sowing of the subsequent maize crop. This shortening is also associated with the adoption of longer-cycle soybean cultivars due to their greater yield stability (Karam et al. 2020; Borghi et al. 2023). Consequently, the success of second-season maize production is increasingly dependent on the adoption of appropriate technologies. These include the selection of adapted hybrids and management practices for earlier sowing, to reduce the risks of delayed planting and to cope with favorable environmental conditions (Salazar et al. 2026). When maize sowing is delayed, crops are frequently exposed to reduced photoperiod and increased risks of frost or drought at the end of the cycle (Karam et al. 2020).

In this context, the Antecipe® system or ‘early intercropped cultivation’ is an alternative strategy to expand the maize sowing window and mitigate climatic risks associated with second-season production (Karam et al. 2020). In this system, maize is sown between soybean rows while the soybean crop is still at the grain-filling stage, 15–20 days before soybean harvest (Silva et al. 2021). This practice can reduce the cycle of the soybean–maize production system by up to 20 days (Karam et al. 2020; Silva et al. 2021). The agronomic and economic advantages of the Antecipe® system include expansion of the maize planting window; reduction of climatic risks associated with late maize sowing; use of longer-cycle soybean cultivars (increasing stability of production); and the expansion of maize cultivation into regions previously considered unfeasible due to the occurrence of frost or drought (Karam et al. 2020).

Despite the numerous benefits associated with the Antecipe® system, the temporal overlap between soybean and maize crops can alter the population dynamics of phytophagous insects (creating a green bridge) and their natural enemies (Dias et al. 2016; Hill et al. 2025). The continuous availability of host plants facilitates the dispersion of pests from senescing soybean to newly established maize seedlings (Panizzi and Bueno 2026). Among the different stink bug species that feed on soybean, the green-bellied stink bug, *Diceraeus melacanthus* (Dallas), has been favored by the adoption of the soybean–maize succession system and turned into one of the main insect pests affecting maize crops (Panizzi et al. 2016, Queiroz et al. 2022, Panizzi and Bueno 2026).

In the Antecipe® system, it remains unclear whether *D. melacanthus* preferentially feeds on senescing soybean plants or on newly emerged maize seedlings, and whether early feeding damage on maize may compromise plant development or the regrowth capacity of maize plants after soybean harvest (when soybean plants are cut close to the soil). Therefore, this study aimed to evaluate the plant preference and damage potential of *D. melacanthus* between maize and soybean plants grown under the Antecipe® system, as well as to assess whether the current economic threshold (ET) for this pest should be reconsidered under this new soybean–maize production system.

## Materials and methods

Two experiments were conducted, one under semi-field (greenhouse) and one under field conditions in Londrina, Paraná, Brazil (23°12′02″S, 51°10′27″W), during the 2024/2025 soybean growing season, to evaluate the host preference between soybean and maize and the damage potential of *D. melacanthus* on maize plants cultivated under the Antecipe® system. In both experiments, the same soybean cultivar, Neo 661 Xtend2 (Cry1Ac + Cry1A.105 + Cry2Ab2), and maize hybrid, AS 1800 PRO3 (Cry1A.105 + Cry2Ab2 + Cry3Bb1), were used.

### Insect rearing

The stink bugs used in this study were from a colony of *D. melacanthus* maintained in the insect laboratory at Embrapa Soybean under controlled conditions (25 ± 2°C, 70 ± 10% RH, and 14L:10D photoperiod), following the methodologies described by Panizzi et al. (2000) and Silva et al. (2008), with minor adaptations. The insects were originally collected from maize fields in Londrina, Paraná, Brazil (23°11′11.7″S; 51°10′46.1″W), during the 2016/2017 growing season, and maintained in a laboratory until the establishment of the experiments with annual introduction of wild stink bugs (from the same farm) to maintain the quality of the insect colony. Adult *D. melacanthus* were kept in plastic cages (20 × 20 × 24 cm) lined with filter paper and raw cotton fabric on the sides as an oviposition substrate.

Insects were provided a natural diet consisting of green bean pods (*Phaseolus vulgaris* L.), soybean seeds (*Glycine max* L.), raw shelled peanuts (*Arachis hypogaea* L.), and sunflower seeds (*Helianthus annuus* L.) (Silva et al. 2008). The cages were cleaned and the food replaced every two days.

Egg masses were collected daily and transferred to smaller plastic containers (11 × 11 × 3.5 cm) lined with filter paper and containing a bean pod as food for the nymphs. When the nymphs reached the fourth instar, they were transferred to the adult containers (plastic cages of 20 × 20 × 24 cm) following the procedures previously described. Insects from this colony (adults of 24–48 h) were used for the experiments or for maintaining the laboratory colony.

### Plant preference of *Diceraeus melacanthus* between soybean and maize under greenhouse simulation of Antecipe® system (experiment 1)

The experiment was conducted in a greenhouse equipped with an automated irrigation system programmed to operate three times daily (08:00, 12:00, and 17:00 h), with irrigation duration adjusted according to the plant development stages. Soybean was sown in individual 5-L pots on June 2, 2025. Once the plants reached the R7 stage (Fehr et al. 1971), maize was sown. Each experimental unit consisted of one soybean plant and one maize plant (in the same pot) maintained together within the same cage. The plants were maintained inside 1.5 × 0.5 m cages made of fine mesh (voile) and equipped with Velcro openings to facilitate evaluations.

The experimental design was completely randomized. In each cage (experimental unit), ten *D. melacanthus* adults were released, allowing free choice between the two host plants. The experiment comprised five cages (experimental units), and the experimental units were repeatedly evaluated over three consecutive days and at three times of the day (8:30, 12:30, and 16:30 h). Infestation was performed on September 24, 2025, when the maize was at the VE stage (seedling emergence and coleoptile rupture at the soil surface; Ritchie et al. 1986). Evaluations were conducted on September 25, 26, and 27, 2025, corresponding to 1, 2, and 3 days after infestation (DAI), respectively. For each combination of evaluation “day” and “time of day,” the number of stink bugs present on each of the soybean and maize plants was recorded to determine the insect’s preferred host.

### Damage levels caused by different densities of *Diceraeus melacanthus* on soybean and maize intercropped in field conditions (Antecipe® system) (experiment 2)

The soybean cultivar was sown on October 18, 2024, with a row spacing of 0.50 m and 15 seeds/m, using standard fertilization for the crop. Herbicide applications were performed on October 29, 2025, using sulfentrazone (500 g + 700 g a.i. ha^−1^; Boral® 500 SC, 250 mL ha^−1^) and glyphosate (662 g a.i. ha^−1^; Preciso XK, 250 mL ha^−1^). Phytosanitary management in the system included applications of the fungicides bixafen + prothioconazole + trifloxystrobin (125 g + 175 g + 150 g a.i. ha^−1^; Fox XPRO®, 0.5 L ha^−1^), mancozeb (750 g a.i. ha^−1^; Unizeb Gold®, 1 kg ha^−1^), and difenoconazole (250 g a.i. ha^−1^; Difenoconazole CCAB 250 EC®, 0.15 L ha^−1^), along with vegetable oil (0.5 L ha^−1^). Both herbicides and fungicides were evenly applied over the entire experiment. Crop monitoring began at the V4 stage using weekly beat cloth sampling to track the main insect pest species, and no insecticide was necessary. When soybean reached the R7 stage (full pods and onset of senescence), maize was planted in the inter-row space of the soybean crop on March 6, 2025, respecting the 0.50 m row spacing corresponding to that used for soybean cultivation. To ensure that the evaluation area was free of stink bugs, an application of imidacloprid + beta-cyfluthrin (100 g + 12.5 g a.i. ha^−1^; Connect®, 1.0 L ha^−1^) was done on February 20, 2025.

The experiment was conducted in 6 × 4 m cages made of fine mesh (voile) (1.8 m tall) with a 1.5 m front zipper for entering the cage to access the plots for evaluations. A completely randomized design was used with four treatments corresponding to *D. melacanthus* densities (zero, one, two, and four adult stink bugs per meter). Four replications were used, each corresponding to one 6 × 4 m cage.

When maize reached the VE stage, manual infestation of stink bugs (of 24–48-h-old adults) inside the cages was performed on March 18, 2025. The densities of one, two, and four insects m^−1^ corresponded to the release of 36, 72, and 144 adults of *D. melacanthus* per cage, respectively, in addition to the control treatment without infestation (0 insects). Damage assessments began on March 24, 2025, six DAI, and were subsequently repeated on March 28 (10 DAI) and 31 (13DAI), 2025. On each date, all plants inside each cage were visually inspected, and a damage score was assigned per plant based on the average level of injury observed, to assess damage progression within each replication/cage. Damage scores were determined using the scale of Roza-Gomes et al. (2011): 0 – Plants without injury; 1 – Leaves with punctures but no size reduction; 2 – Plants with slight whorl injury, with size reduction (slightly curled); 3 – Plants with a rotten whorl or tillering; 4 – Plants with a dry or dead whorl.

Maize was harvested manually. For yield evaluation, ears were collected and counted from four 5-m rows within each experimental unit. Grain yield (kg ha^−1^) was determined after mechanical and manual threshing of the harvested ears, followed by grain weighing, with moisture content corrected to 13%.

### Statistical analyses

Preference data were analyzed using a Generalized Linear Mixed Model (GLMM) with a binomial distribution and logit link function. Plant species (soybean vs maize), day of evaluation (1, 2, and 3 DAI), and time of day (8:30, 12:30, and 16:30 h) were included as fixed effects, together with the plant species × day and plant species × time-of-day interactions. Cage identity was included as a random effect to account for the non-independence of repeated observations within the same experimental unit. Damage level data were analyzed using GLMM with a beta-binomial distribution and logit link function, including stink bug density, evaluation time, and their interaction. Models were fitted using the *glmmTMB* package (Brooks et al. 2017), and adjusted means and multiple comparisons were obtained using the *emmeans* package (Lenth 2020), with Tukey adjustment (α = 0.05). Yield data (kg ha^−1^) were subjected to residual normality verification using the Shapiro–Wilk test (Shapiro and Wilk 1965) and homogeneity of variances using Levene’s test (Levene 1960). When model assumptions were met, analysis of variance (ANOVA) was performed and, when significant effects were detected (*p* < 0.05), means were compared using Tukey’s test (1949). All statistical analyses were performed using the R statistical software (R Core Team 2024).

## Results

### Preference of *Diceraeus melacanthus* between soybean and maize under the Antecipe® system (experiment 1)

**Fig. 1:**
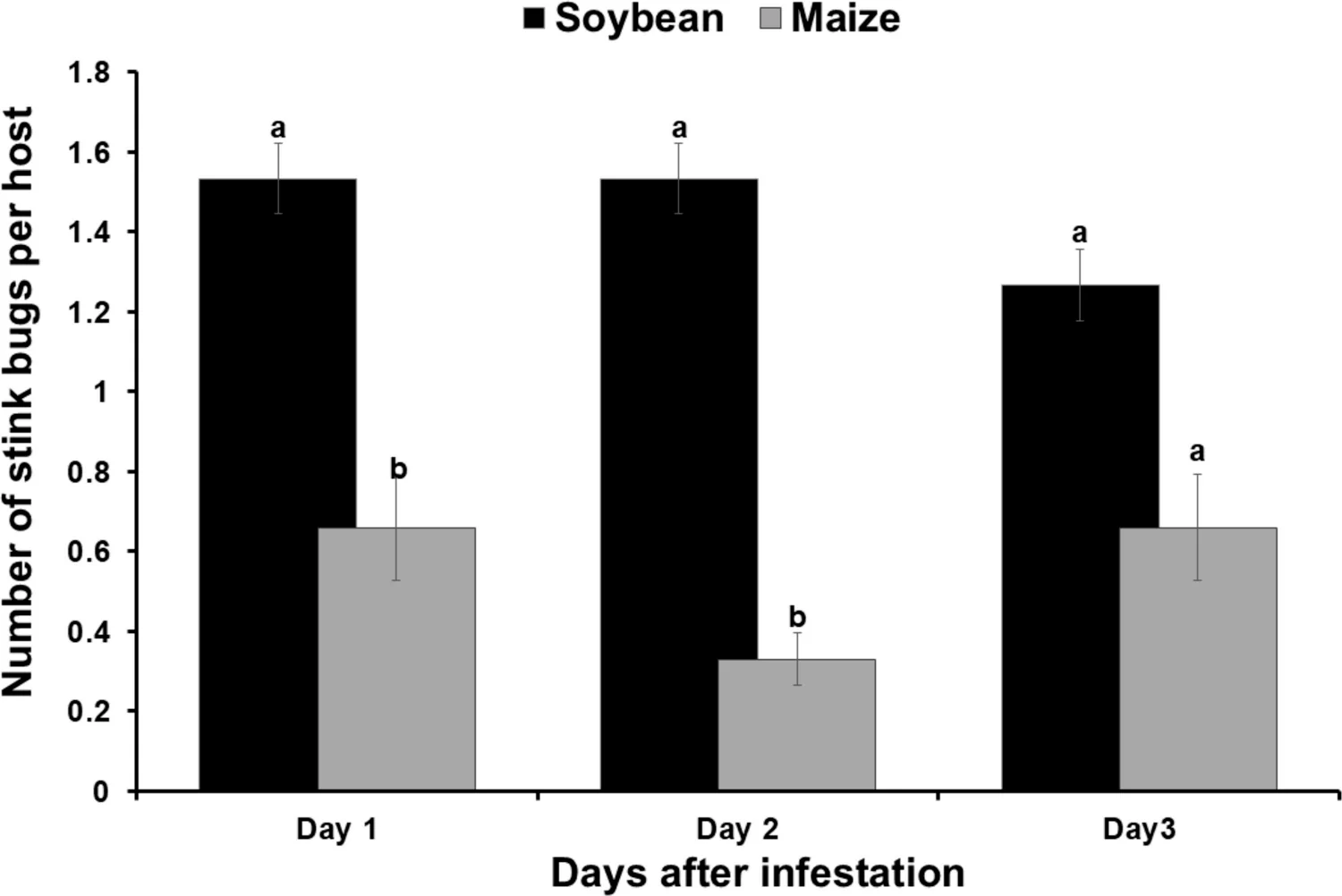
Number of adult *Diceraeus melacanthus* (Dallas) per host (soybean and maize) (Mean ± SEM of the day) on different days after insect infestation in the dual-choice test conducted under semi-field conditions (experiment 1). Different letters within each day of evaluation indicate significant differences between hosts based on generalized linear mixed models (GLMM), followed by pairwise comparisons using the Tukey test (*p* < 0.05)

The preference of *D. melacanthus* differed significantly between the two crops (χ^2^ = 23.82, df = 1, *p* < 0.001), with a greater number of adults recorded on soybean plants across all evaluation days, although this was not statistically different on day 3 (Fig. 1).

**Fig. 2:**
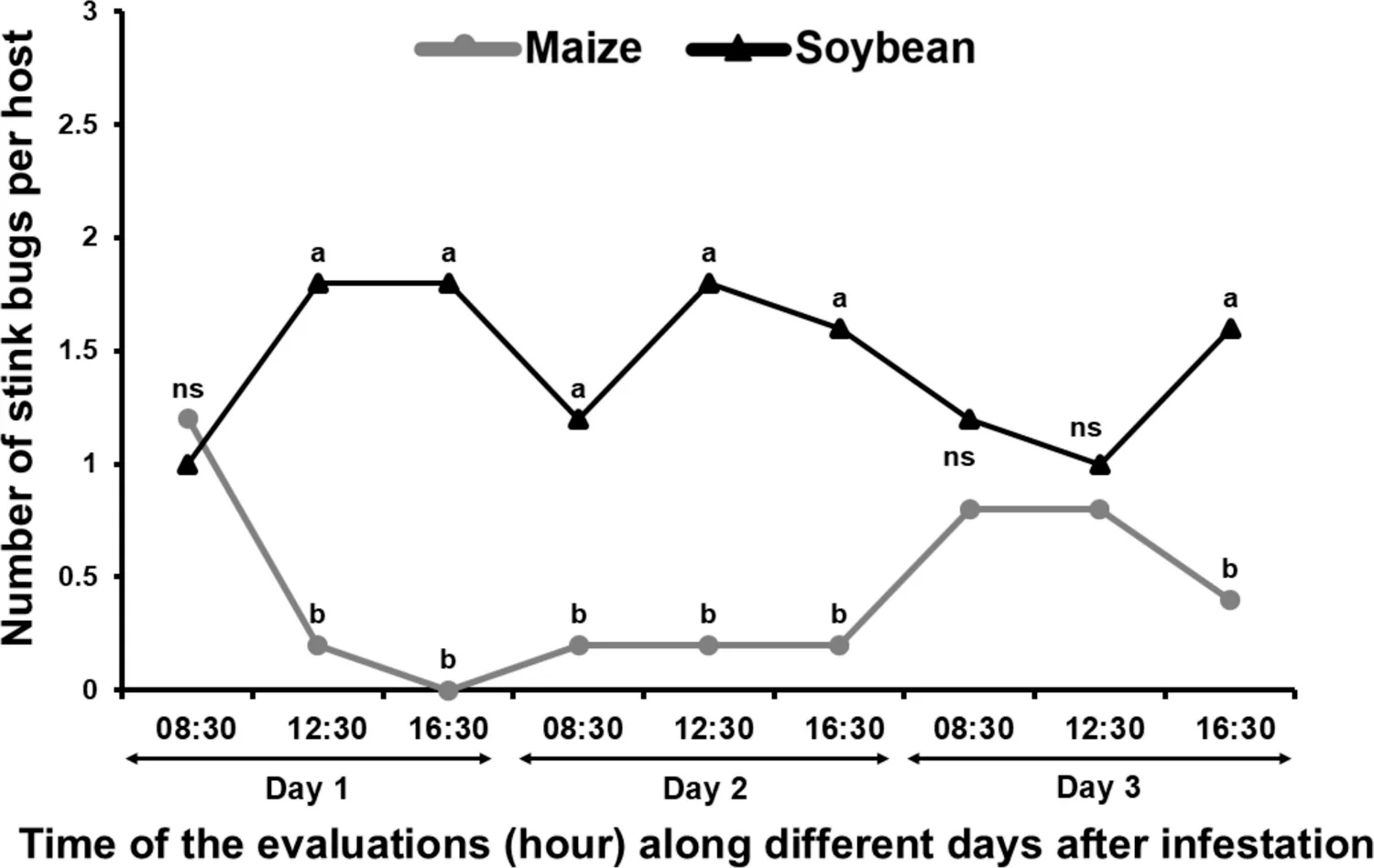
Mean number of adult *Diceraeus melacanthus* (Dallas) recorded on each host (soybean and maize) along the days after infestation at different hours of the day (experiment 1). Different letters within each day and time indicate significant differences between hosts for each evaluation, based on generalized linear mixed models (GLMM), followed by pairwise comparisons using the Tukey test (*p* ≤ 0.05). ns: Non-significant results are indicated as (*p* > 0.05)

Host plant choice was also significantly affected by evaluation time (χ^2^ = 68.12, df = 2, *p* < 0.001). On soybean, stink bug activity was usually highest during the warmest hours (12:30 and 16:30 h, except 12:30 of day 3), with mean values above 1.5 individuals per plant. However, the differences in the time of day on maize were not clear, and infestation was usually at lower densities than soybean (Fig. 2).

The interaction between host and time of evaluation was significant (χ^2^ = 11.64; df = 2; *p* < 0.003), indicating that differences between crops varied throughout the day (Fig. 2). Soybean consistently showed higher mean numbers of insects across all evaluation times, ranging from 1.26 ± 0.18 to 1.53 ± 0.27 adults per plant. Maize presented lower values, varying from 0.20 ± 0.11 to 0.66 ± 0.18 adults per plant (except first evaluation at 8:30 on day 1). The difference between crops decreased on the third evaluation day, when maize reached its highest mean value (0.67 ± 0.22).

During the cooler morning period (8:30 h), stink bugs were usually more evenly distributed between both host plants. In contrast, beginning at 12:30 h, a clear divergence between the curves was observed, with significantly higher densities recorded on soybean during the warmer period of the day. This pattern was consistent across the three evaluation days, although the magnitude of the response varied among days (Fig. 2).

### Damage caused by *Diceraeus melacanthus* on soybean and maize intercropped in the Antecipe® system (experiment 2)

**Fig. 3:**
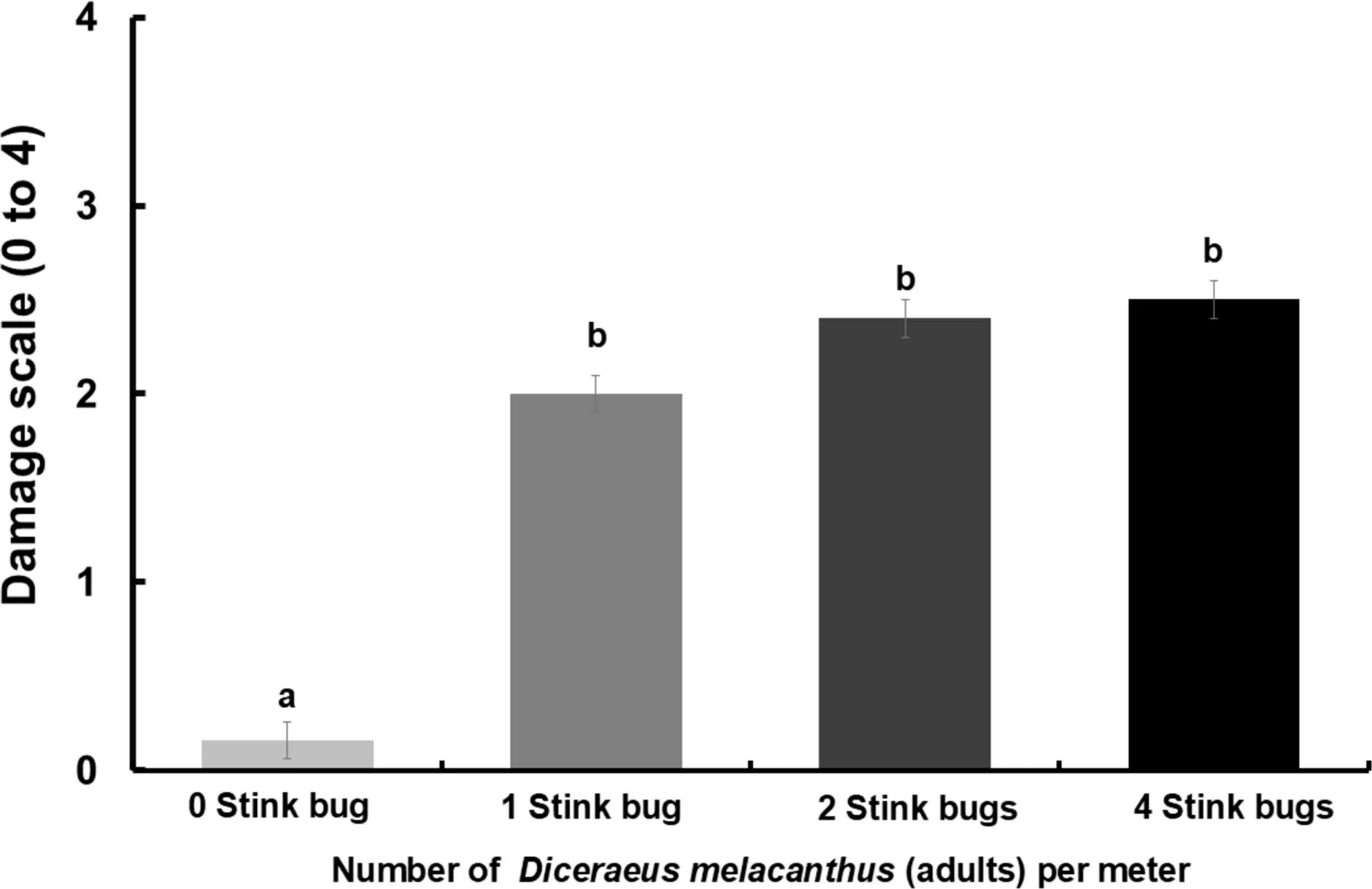
Damage levels (Mean ± SEM) in maize, assessed using the scale proposed by Roza-Gomes et al. (2011) [average of 3 days of evaluation (6, 10, and 13 days after infestation) of all plants in the cage] (experiment 2). Different letters indicate significant differences among densities based on a generalized linear mixed model (GLMM) followed by Tukey’s post hoc test (*p* ≤ 0.05)

Increasing densities of *D. melacanthus* resulted in progressively higher damage levels in maize plants (χ^2^ = 32.44; df = 3; *p* < 0.001), although with no statistical difference among treatments with 1, 2, and 4 stink bugs/m. The lowest damage was recorded in the control treatment (0 stink bugs), with mean values close to zero. At densities of 1, 2, and 4 stink bugs/m, mean damage scores were 2.0, 2.4, and 2.5, respectively (Fig. 3).

**Fig. 4:**
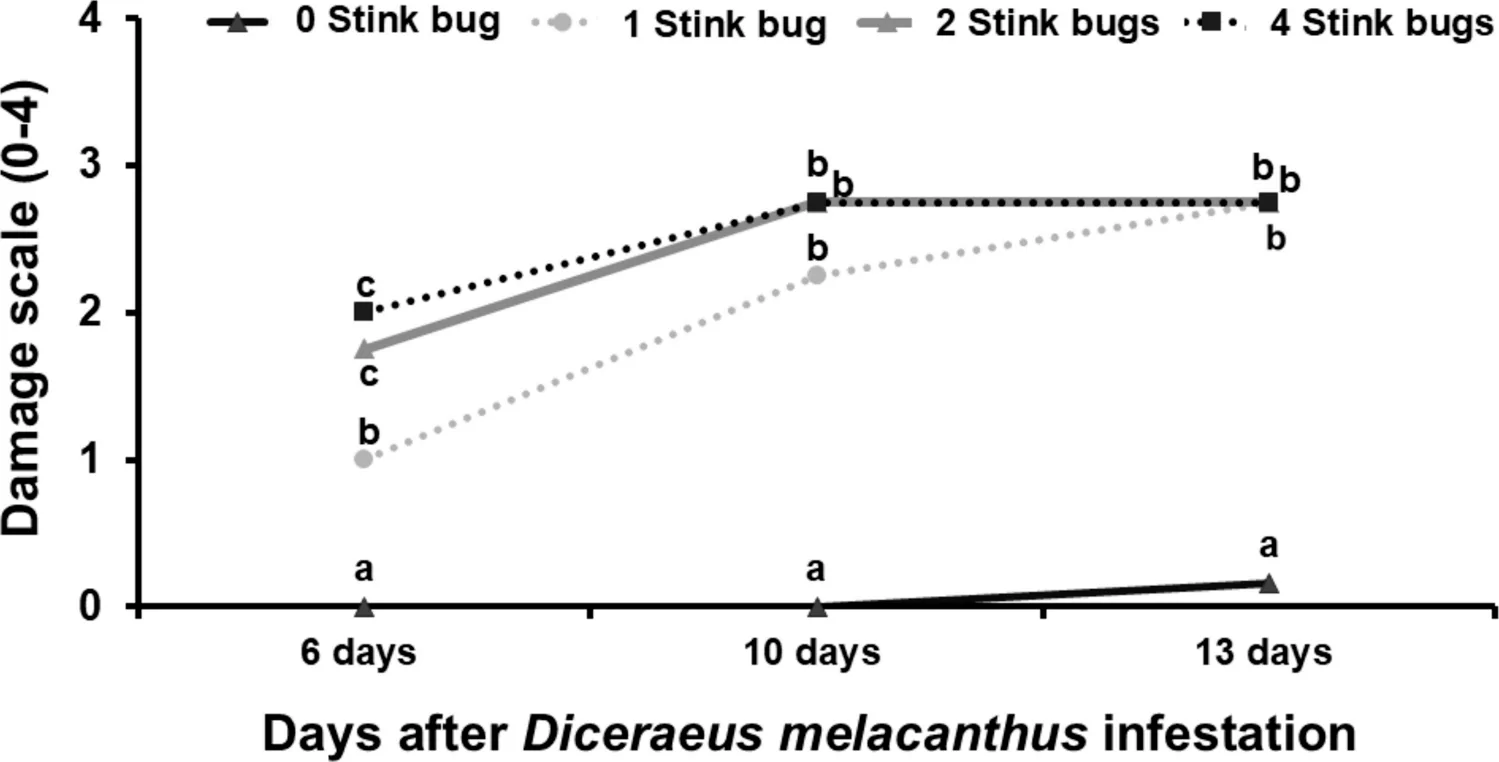
Damage levels (Mean ± SEM) in maize assessed using the scale of Roza-Gomes et al. (2011) on different days after field-cage infestation of *Diceraeus melacanthus* (Dallas) with 0, 1, 2, and 4 stink bugs per meter (mean per day of all plants of the whole cage) (experiment 2). Different letters within each day of evaluation indicate differences among stink bug densities (GLMM, Tukey’s test, *p* ≤ 0.05)

Damage levels in maize plants increased over the evaluation period (χ^2^ = 45.19, df = 3, *p* < 0.001; Fig. 4). At six DAI, damage scores ranged from 1.0 at the density of 1 stink bug/m to 2.0 at 4 stink bugs/m. By ten DAI, all infested treatments with stink bugs exceeded a damage score of 2.0, with mean values of 2.25, 2.75, and 2.75 recorded at densities of 1, 2, and 4 stink bugs/m, respectively. At thirteen DAI, the damage score at the lowest density (1 stink bug/m) reached 2.75, comparable to the levels observed in the higher-density treatments.

The interaction between density and time was not significant (p > 0.158), indicating that the rate of damage progression was similar across infestation levels; however, initial damage scores were higher at greater densities. Overall, the results demonstrate that at a density of 1 stink bug/m, the mean damage score more than doubled that of the control, while at 2 stink bugs/m, damage reached levels near the maximum score before plant death (2.75).

**Fig. 5:**
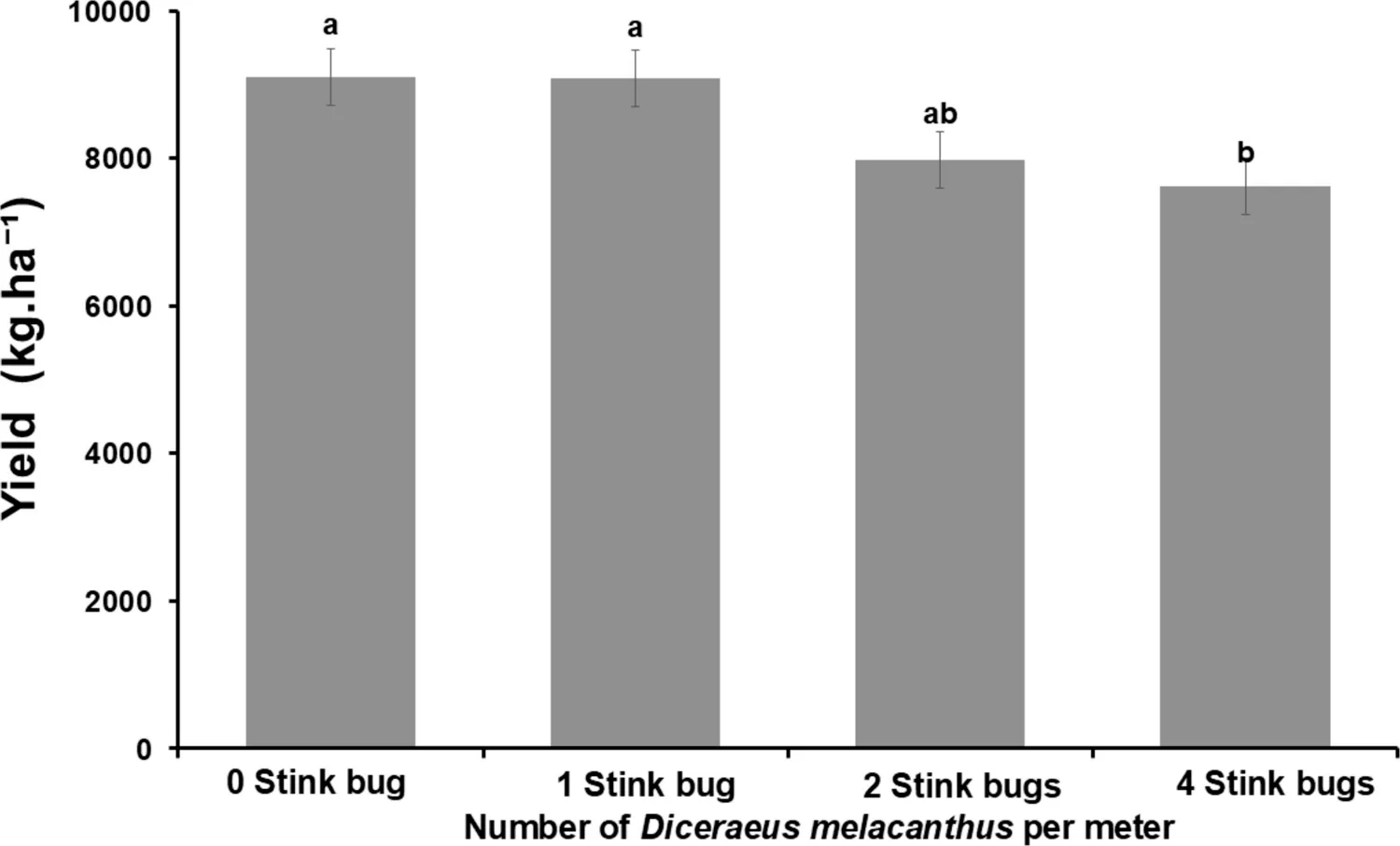
Maize yield (Mean ± SEM) under different infestation densities of *Diceraeus melacanthus* adults per meter inside field cages in the Antecipe® system (experiment 2). Different letters indicate significant differences among treatments (Tukey’s test, *p* ≤ 0.05)

Grain yield was significantly affected by stink bug density (F₃,₁₂ = 3.705; *p* = 0.044; Fig. 5). Mean values ranged from 9,108.9 kg ha^−1^ (151.8 bags ha^−1^) in the non-infested control to 7,615.0 kg ha^−1^ (126.9 bags ha^−1^) at the highest density (4 stink bugs/m), corresponding to a reduction of 1,494 kg ha^−1^ (16.4%). Pairwise comparisons indicated that grain yield at 4 stink bugs/m was significantly lower than at 0 (*p* = 0.022) and 1 stink bug/m (*p* = 0.035), whereas all other pairwise comparisons were not significant (*p* > 0.05). The treatment with 2 stink bugs/m showed intermediate yield (7,979.6 kg ha^−1^; 133 bags ha^−1^) and did not differ significantly from either the lower-density treatments (0 and 1 stink bug/m) or from the highest-density treatment (4 stink bugs/m).

## Discussion

These results reinforce the impact of *D. melacanthus* as a key pest of the stink bug complex in soybean–maize succession systems, particularly under the Antecipe® system. The phenological overlap between crops ensures continuous host availability, leading to the persistence and feeding activity of this species (Borghi et al. 2023; Panizzi and Bueno 2026).

Although *D. melacanthus* is commonly described as a species preferentially associated with grasses, especially maize (Corrêa-Ferreira and Sosa-Gómez 2017), under the conditions of this study, an initial preference for soybean plants was observed. This preference could have been because soybean grains were used to rear the nymphs before the experiments with adults. As a hemimetabolous insect, pre-imaginal conditioning can be triggered in Pentatomidae from the final nymphal instar to the adult stages, orienting adults’ feeding choices (Panizzi 1997). In addition, both the quantity and the quality of food consumed by insects have strong effects on pest behavior (Panizzi and Parra 1991). The most nutritiously favorable plants are usually preferred by insects (Bernays and Chapman 1994). A previous study has reported feeding preference of *D. furcatus* to soybean over wheat as well as feeding preference of *Euschistus heros* (F.) to soybean over both wheat and canola (Possebom et al. 2020), illustrating that the soybean has high nutritional quality for Pentatomidae development (Queiroz et al. 2022) what can have impacted the feeding preference results.

The fluctuation in stink bug occurrence between soybean and maize throughout the day indicates a dynamic host-use behavior (Panizzi and Lucini 2024), with greater presence on maize during periods of milder temperatures, which are associated with feeding activity on maize. In contrast, during the warmer periods, higher activity was observed on soybean, which may act as shelter (especially from the sun) and thermal refuge, offering a cooler and more humid microhabitat. Soybean canopy at the R7 developmental stage is considerably higher and denser than maize seedlings at emergence. Consequently, the continuous evapotranspiration from soybean leaves, combined with restricted air circulation within large, closed canopies, significantly increases internal humidity, creating an environment that is much more humid and cooler than the exterior (Körner 2021). Such different environmental conditions, especially in temperature and relative humidity, can impact the distribution of the stink bug population inside the plant canopy (Owens et al.^2013^) as each plant microclimate is influenced by plant canopy height and density (Grisafi et al. 2021). Stink bugs can suffer desiccation in low-humidity environments (Owens et al.^2013^) and suppression of their gut symbionts due to high temperatures (Muluvhahothe et al. 2024), reducing the survival of nymphs and adults.

In the Antecipe® system, soybean may function as a support host, allowing *D. melacanthus* to remain active in the agroecosystem precisely when maize is emerging and at its most vulnerable stage (Silva et al. 2019; Fernandes et al. 2020). This pattern is particularly relevant when considered together with soybean harvest in the Antecipe® system, which often causes mechanical damage to maize seedlings and temporarily reduces leaf area (Nielsen 2019; Karam et al. 2020; Borghi et al. 2021). Although maize plants can recover from mechanical damage at early stages, especially when the apical meristem is not completely compromised and plant stand is maintained (Magalhães and Durães 2006; Borghi et al. 2021), injuries to the whorl and remaining tissues may increase physiological stress and limit the plant’s compensatory response, increasing the likelihood of yield losses (Santos et al. 2025). In addition, previous studies have shown that when maize plants are subjected to mechanical damage, infestation by *D. melacanthus* reduces plant height, stem diameter, and biomass accumulation. Severe defoliation further increases plant susceptibility to stink bug injury, particularly when infestation occurs after mechanical damage (Santos et al. 2025).

In the field experiment, the positive relationship between population density and damage intensity reflects the efficient feeding behavior of *D. melacanthus* on young tissues, characterized by deep stylet penetration and the injection of phytotoxic saliva, resulting in deformation, whorl death, and reduced plant vigor (Corrêa-Ferreira and Sosa-Gómez 2017). Even low population densities caused substantial injury to maize, highlighting the high phytotoxic potential of the green-bellied stink bug, consistent with previous reports on the effects of salivary enzymes and early physiological impairment of maize seedlings (Roza-Gomes et al. 2011; Bortolotto et al. 2022; Castilhos et al. 2026).

By demonstrating the rapid progression of injury scores, the transition from soybean to maize, and the limits of maize compensatory capacity under combined stress conditions, this study contributes to the understanding of phytosanitary risks in intensified succession systems. This scenario becomes even more critical considering that field-scale studies have shown that late-season control strategies in soybean, such as insecticide applications at the end of the soybean crop cycle or pre-harvest desiccation, are ineffective in reducing subsequent infestations of *D. melacanthus* in maize (Queiroz et al. 2025). These findings indicate that the risk is not limited to active pest movement after harvest but is primarily associated with the spatial and temporal continuity of host plants, a condition intensified in the Antecipe® system, and significantly reduces the margin for decision-making in pest management.

These results provide critical insights for refining established ETs within the specific context of the Antecipe® system. In the present study, densities of 2 stink bugs/m caused intermediary damage with recorded yields similar to treatments with both 1 and 4 stink bugs/m. A significant reduction in grain yield relative to the non-infested control was only detected at the highest density tested (4 stink bugs/m). These results suggest that 2 stink bugs/m is the turning point from plant tolerance to plant compensation in the damage curve response proposed by Higley and Peterson (1996). Therefore, considering that 3 *D. melacanthus*/m is proposed as ET by Gomes et al. (2020) for maize sowed in the second season after soybean (not considering the Antecipe® system), in addition to the cumulative stress from stink bugs injuries associated with mechanical injuries during soybean harvest in the Antecipe® system, it is reasonable to propose that 2 stink bugs/m should be adopted as a more conservative (yet preliminary) ET for maize cultivated in this system. Such ET must now be validated in farm conditions, under different climatic conditions (different regions), with different maize varieties.

As previously discussed, under the Antecipe® system, factors such as mechanized soybean harvesting can inflict additional mechanical damage on maize seedlings, temporarily reducing functional leaf area (Santos et al. 2025). This cumulative stress likely diminishes the crop’s compensatory capacity, increasing its sensitivity to *D. melacanthus* feeding during early developmental stages. Consequently, population densities that might not result in immediate economic losses in conventional systems may gain greater significance under the Early Intercropped Cultivation model, justifying a more conservative ET of two stink bugs/m. From an operational standpoint, however, adopting this ET still presents practical challenges.

Since maize is established when soybean is in advanced reproductive stages (R6–R7), with a maximum of 20 days of anticipation, the window for insecticide application is narrow and must strictly adhere to pre-harvest intervals (PHI) to avoid residues in soybean grains (Queiroz et al. 2025). Nevertheless, within this critical window, detecting populations ≥ 2 insects/m, especially of *D. melacanthus*, may signal the need for early intervention to prevent successive attacks on maize after the soybean harvest. Such management must prioritize frequent and early monitoring to ensure that chemical control is viable before the safety interval for harvest is exceeded. Maize seed treatment for stink bug control can be adopted in addition to the sowing of more tolerant maize cultivars (with faster growing and higher diameter of cornstalk), as proposed by Sutil et al. (2026).

In conclusion, results reinforce that, under the Antecipe® system, management of the green-bellied stink bug should be planned in an integrated manner, considering soybean phenology, maize establishment timing, and the legal and operational constraints associated with insecticide use. Although the high injury scores observed in this study indicate substantial damage potential, actual yield losses may depend on infestation duration, population density, and the crop growth stage at which the attack occurs. Note that the data were obtained under controlled conditions (cages); therefore, further validation under open-field conditions is required to confirm the applicability of the proposed economic threshold on the larger farm scales.

## Supplementary Information

Below is the link to the electronic supplementary material.

Supplementary file1 (XLSX 15.7 KB)

## Data Availability

Raw data is available as supplemental material of this article.
